# Association of Severity of Coronary Lesions with Bone Mineral Density in Postmenopausal Women

**DOI:** 10.5935/abc.20180035

**Published:** 2018-03

**Authors:** Rui Xu, Xin-Chun Cheng, Yuan Zhang, Hong-Mei Lai, Hong-Ni Yang

**Affiliations:** 1 Gerontology Center - People’s Hospital of Xinjiang Uygur Autonomous Region, Urumqi, Xinjiang - China; 2 Department of Pacing and Electrophysiological - The First Affiliated Hospital of Xinjiang Medical University, Urumqi, Xinjiang - China; 3 Department of Cardiology - People’s Hospital of Xinjiang Uygur Autonomous Region, Urumqi, Xinjiang - China

**Keywords:** Coronary Artery Disease, Osteoporosis, Postmenopausal, Bone Density, Stroke, Morbidity, Bone Diseases, Metabolic

## Abstract

**Background:**

Coronary artery disease (CAD) and osteoporosis (OP) are common diseases in postmenopausal women. In both cross-sectional and longitudinal epidemiologic studies, low bone mass has been related to increased frequency of CAD. However, available data on the relationship between bone mineral density (BMD) and severity of coronary lesions is limited.

**Objective:**

To investigate association between the BMD and severity of coronary lesions assessed by Gensini score in postmenopausal women.

**Methods:**

This study included 122 postmenopausal women who were diagnosed with CAD. These patients were divided into two groups according to the severity of coronary lesions assessed by the Gensini score - patients with mild coronary lesions (Gensini score < 25) and patients with severe coronary lesions (Gensini score ≥ 25). Femoral neck mineral density was measured with dual energy X-ray absorptiometry (DXA).

**Results:**

The study included postmenopausal women aged 64.31 ± 4.71 years, 85 of whom (69.7%) exhibited severe coronary lesions. Participants with severe coronary lesions had a significantly higher T score than did those with mild coronary lesions at the femoral neck (p < 0.05). The mean T-score was −0.84 ± 1.01 in mild coronary lesions group, −1.42 ± 1.39 in severe coronary lesions group (p < 0.05). Multivariable logistic regression analysis showed that osteopenia-osteoporosis at the Femoral neck (odds ratio 2.73; 95% confidence interval 1.06 to 6.13) was associated with an increased risk of developing severe coronary lesions. The multiple regression model showed that T-scores (b = −0.407, SE = 0.151, p=0.007) were the independent predictors of Gensini score.

**Conclusion:**

The relationship between severity of coronary lesions and BMD was significant in postmenopausal women. BMD, a low-cost technique involving minimal radiation exposure, widely used for osteoporosis screening, is a promising marker of severity of coronary lesions.

## Introduction

Atherosclerosis (AS) is one of the most common diseases in elderly people, especially in postmenopausal women. The complications of AS, like coronary artery disease (CAD) and cerebrovascular diseases reduce quality of life and lead to excess morbidity.^1^ Epidemiology studies found that the CAD morbidity and mortality rates were significantly higher in postmenopausal women compared with premenopausal women.^2^ Unlike younger women, the risk of CAD in older women is higher when there is a decrease in estrogen production, marking the end of the protective effect of endogenous estrogens against CAD.^3-5^ Therefore, identifying the risk factors associated with CAD in postmenopausal women is critical for improving patients' survival rate and life quality.

Recently, increasing evidence has accumulated to support the correlation between low bone mineral density (BMD) and AS.^6-8^ AS and osteopenia-osteoporosis syndrome share some risk factors, among which are parathyroid hormone, lack of estrogen, homocysteine, inflammatory process, vitamins D and K, lipid oxidation products, molecular pathways involved in bone and vascular mineralization, and calcification mechanisms that seem to be similar in vascular structure and bone.^9,10^

We have previously reported that coronary artery calcium scores, an earlier sign of coronary artery AS, were significantly higher in the osteopenia/osteoporosis groups compared to normal BMD groups, and that these values were negatively associated with T-scores. These findings indicate that decreased BMD may increase the risk of CAD.^11^ However, little is known about the association between decreased BMD and severity of coronary lesions in postmenopausal woman.

Therefore, the aim of this cross-sectional study was to examine the associations between BMD and coronary lesions assessed by Gensini score in postmenopausal women who attended our Laboratory and who had their BMD measured and grouped by the severity of CAD.

## Methods

### Study population

A total of 122 female patients who were admitted to the cardiology clinic with chest pain between January 2014 and August 2016 were included in the study. Inclusion criteria were postmenopausal women aged ≥ 50 years, who were diagnosed with acute coronary syndrome or chronic CAD. This diagnosis was made by history of angina pectoris or myocardial infarction, electrocardiographic findings, cardiac enzymes, and coronary angiography results. These patients underwent a bone densitometry on a routine basis within the previous 12 months, and were not taking any medication with known effect on bone turnover. Exclusion criteria were patients with normal coronary angiography; patients who had moderate-to-severe heart valve disease and decompensated heart failure; patients with severe kidney or liver failure, malignancy, hematological diseases, or autoimmune disorder.

### Clinical features and laboratory examination

The weight and height were measured at each of eligible patient. Body mass index (BMI) was calculated as body weight/height^2^ (kg/m^2^). Information concerning the history of diseases (diabetes, hypertension, and hyperlipidemia) was collected using a standard questionnaire.

Hypertension was defined as history of hypertension and/or an average systolic blood pressure (SBP) ≥ 140 mmHg and/or an average diastolic blood pressure (DBP) ≥ 90 mmHg on two separate occasions. Diabetes was defined as history or presence of diabetes and/or a fasting plasma glucose level > 126 mg/dL on 2 separate occasions, or a random glucose value of > 200 mg/dL on one or more occasion. Hypercholesterolemia was defined as a total serum cholesterol level of > 240 mg/dL; high triglyceride (TG) and high LDL-cholesterol (LDL-c) were defined as total serum TG > 200 mg/dL and LDL-C > 160 mg/dL, respectively.

### BMD measurement

Participants had undergone a BMD test of the left femoral neck bone by dual-energy X-ray absorptiometry using a QDR 4500A fan beam bone densitometer (Bedford, MA, USA) according to the manufacturer’s instructions within the previous 12 months. BMD results were reported as T-scores, which were also categorized into three groups according to the World Health Organization (WHO) criteria for diagnosing osteoporosis: normal BMD (T-score≥-1 SD); osteopenia (T < -1 SD and > -2.5 SD); and osteoporosis (T-score ≤ -2.5 SD).^11^

### Gensini risk scoring

Coronary angiography was performed in all subjects. Gensini score: angiographic stenosis of a culprit artery in the range of 0% to 25% was scored as 1 point, stenosis in the range of 25% to 50% was scored as 2 points, 50% to 75% was scored as 4 points, 75% to 90% was scored as 8 points, 90% to 99% was scored as 16 points, and total occlusion was scored as 32 points. A multiplier was assigned to each vascular segment based on the functional significance of the myocardial area supplied by that segment: 5 for the left main coronary artery, 2.5 for the proximal segment of the left anterior descending (LAD) coronary artery and the proximal segment of the circumflex artery, 1.5 for the mid-segment of the LAD, 1.0 for the right coronary artery, the distal segment of the LAD, the mid-distal region of the circumflex artery, the posterolateral artery, and the obtuse marginal artery, and 0.5 for other segments.^12^ Angiographic evaluations were reviewed by the consensus of two observers with more than two years of experience. Based on the Gensini score, patients were divided into two groups - 37 patients in the group of mild coronary lesions (Gensini score < 25 points) and 85 patients in the group of severe coronary lesions (Gensini score ≥ 25 points); this grouping was compatible with the literature.^13^

### Statistical analyses

Analyses were carried out using SPSS version 17.0 (SPSS Inc., Chicago, IL). Continuous variables with a Gaussian distribution are presented as mean ± standard deviation (SD), and those with a non-Gaussian distribution are presented as median values with corresponding 25th and 75th percentiles. The normal distribution of different parameters was verified with the Kolmogorov-Smirnov test. Differences between the groups were evaluated using unpaired t-test or the Mann-Whitney U-test. Categorical variables were compared with the chi-square test or Fisher’s exact test (Fisher’s exact test was used for frequencies of osteoporosis in Table 2). The association between BMD and risk for severe coronary lesions was evaluated by multiple logistic regression analysis. Multiple linear regression analysis was performed to assess whether BMD was the independent explanatory factor for the severity of coronary lesions (assessed by the Gensini score) in postmenopausal women. Statistical significance was set at p < 0.05 (2-tailed).

**Table 2:** Comparison of clinical parameters between the groups with mild coronary lesions and severe coronary lesions

Parameter	Mild coronary lesions group	Severe coronary lesions group	p value
	Gensini score < 25	Gensini score ≥ 25	
	n = 37	n = 85	
Age (years)	62.33 ± 5.65	65.17 ± 4.43	0.003
Body mass index (kg/m^2^)	26.23 ± 2.53	26.17 ± 2.47	0.872
Hypertension, n (%)	13 (35.1%)	35(41.2%)	0.530
Diabetes, n (%)	9 (24.3%)	38 (44.7%)	0.034
Hyperlipidemia, n (%)	11 (29.7%)	27(31.8%)	0.824
T-score	-0.84 ± 1.01	-1.42 ± 1.39	0.024
osteoporosis, n (%)	3(8.1%)	21 (24.7%)	0.034
osteopenia, n (%)	10 (27.0%)	41 (48.2%)	0.029
osteoporosis or osteopenia, n (%)	13 (35.1%)	62 (72.9%)	0.000

Continuous variables with non-Gaussian distribution (except for those expressed as median) were compared using t-tests. For values expressed as median (25th and 75th percentiles), P values were determined by Mann-Whitney U test. Categorical variables were compared by chi-square test, except for osteoporosis, which were compared by Fisher's exact test (expected frequencies of ≤ 5).

## Results

A total of 122 postmenopausal women (mean age 64.31 ± 4.71) were included in the present study, 69.7% of whom exhibited severe coronary lesions. Clinical characteristics of all participants at baseline are summarized in Table 1. In all, 19.6% of the patients were found to have osteoporosis in the femoral neck and 41.8% osteopenia; 39.3% of the women suffered from high blood pressure, 38.5% have diabetes, and 31.1% have hyperlipidemia.

**Table 1:** Characteristics of the study population (n = 122)

Age (years)	64.31 ± 4.71
Body mass index (kg/m^2^)	26.19 ± 2.49
Hypertension, n (%)	48 (39.3%)
Diabetes, n (%)	47 (38.5%)
Hyperlipidemia, n (%)	38 (31.1%)
T-score	-1.24 ± 1.27
Gensini score	43.46 (17.5, 73)
osteoporosis, n (%)	24 (19.6%)
osteopenia, n (%)	51 (41.8%)
osteoporosis or osteopenia, n (%)	75 (61.5%)

Continuous variables with a Gaussian distribution are presented as mean ± SD, and those with a non-Gaussian distribution are presented as median values with corresponding 25th and 75th percentiles. Categorical data are expressed as absolute numbers with (percentages).

Table 2 shows the comparison between the groups with mild coronary lesions and severe coronary lesions in terms of some clinical parameters. Patients with severe coronary lesions patients were older, and had higher prevalence of diabetes and osteoporosis/osteopenia compared with those with mild coronary lesions (p < 0.05). There were no differences between the groups with respect to BMI, proportions of patients with hypertension and hyperlipidemia.

Univariate logistic regression analysis showed that osteoporosis/osteopenia was a risk factor for severe coronary lesions (OR = 2.51, 95% CI, 1.153-5.657, p = 0.003). Corresponding to these findings, multivariate logistic regression analysis was used to detect the association between osteoporosis/osteopenia and risk of severe coronary lesions. After adjusting for confounding factors such as age, hypertension, diabetes, and hyperlipidemia, the osteoporosis /osteopenia remained the risk factors for severe coronary lesions (OR = 2.73, 95% CI, 1.06-6.13, p = 0.007, Table 3).

**Table 3:** Adjusted odds ratio of risk factors for severe coronary lesions

Independent variable	Odds ratio (95%CI)	p value
Osteopenia or osteoporosis	2.73(1.06-6.13)	0.007
Age	1.24(1.19-2.65)	< 0.001
BMI	1.37(0.73-3.57)	0.706
Hypertension	2.31(0.83-5.31)	0.313
Diabetes	3.13(0.96-7.37)	0.082
Hyperlipidemia	1.39(0.57-3.62)	0.431

BMI: body mass index; 95% CI: 95% confidence interval

When Gensini score was considered as the dependent variable in a linear regression model, T-score (b = −0.407, *SE*= 0.151, p = 0.007) and age (b = 0.295, *SE*= 0.132, *P*= 0.023), but not diabetes, hypertension, BMI, and hyperlipidemia, were the independent predictors of Gensini score.

In a linear regression analysis with Gensini score as a dependent variable and age, T-score, diabetes, hypertension, BMI, and hyperlipidemia as independent variables (Table 4), only T-score (b = −0.407, *SE*= 0.151, p = 0.007) and age (b = 0.295, *SE*= 0.132, p = 0.023) correlated with Gensini score.

**Table 4:** Multiple regression analysis of Gensini score (dependent variable) versus age, diabetes, hypertension, body mass index, hyperlipidemia, and T-score (independent variables).

Independent variable	β	SE	p value
T-score	0.407	0.151	0.007
Age	0.295	0.132	0.023
Body mass index	0.183	0.203	0.136
Hypertension	0.147	0.134	0.254
Diabetes	0.113	0.179	0.572
Hyperlipidemia	0.053	0.121	0.697
*R*^2^		0.31	

β: Values are standardized coefficient; SE: values are standard error of β. R^2^: values are the total explained variance of the model.

## Discussion

In our study, postmenopausal women with severe coronary lesions are more likely to have osteopenia/osteoporosis compared with mild coronary lesions group, independent of other risk factors. This suggests that postmenopausal women with osteopenia/osteoporosis may have a higher risk of developing severe coronary lesions. Our findings are in accordance with previous studies demonstrating the relationship between BMD and CAD that concluded that BMD is a promising marker of severity of CAD.

Both osteopenia and AS are serious public health problems that can threaten people's health and quality of life.^14,15^ Previous studies have proved a clear link between AS and BMD. In a retrospective study including 1,335 elderly patients, the incidence of CAD increased in low BMD patients, compared with patients with normal BMD. Multiple logistic regression analysis confirmed that low BMD is associated with CAD, after adjustment for diabetes mellitus, hypertension, smoking, and age.^16^ Another study with 252 postmenopausal women showed that osteopenia/osteoporosis at the lumbar spine or femoral neck was associated with coronary AS assessed by 64-row multidetector computed tomography.^17^ Our previous study showed that another measure of AS, coronary artery calcification, was associated with BMD of the lumbar spine in healthy postmenopausal women. The odds for coronary artery calcification in osteoporotic women were over three-fold higher compared with those in women with a normal BMD.^11^

Gensini score is an important angiographic scoring system used to assess the extent, severity, and complexity of CAD. CAD patients with high Gensini score are more likely to report major adverse cardiac events. Therefore, identifying CAD patients with high Gensini scores is critical for reducing CAD-related disability and death.^18,19^ There are a few studies about the relationship between BMD and severity of coronary lesions. A retrospective study carried out with 55 male patients with CAD, confirmed by coronary angiography, showed that decreased BMD was associated with severe coronary lesions assessed by Gensini score, independent of other cardiovascular risk factors.^13^ Similarly, a study involving 74 male CAD patients revealed that the incidence of osteopenia/osteoporosis in severe coronary artery lesions group determined by SYNTAX score was significantly higher than mild coronary artery lesions group.^20^ However, most of these studies have been based on male CAD patients while few studies have involved postmenopausal, CAD women patients. In our study, 186 postmenopausal women with CAD patients identified by coronary angiography were divided into two groups by Gensini scoring: mild coronary lesions patients (Gensini score < 25) and severe coronary lesions patients (Gensini score > 25). We found that there was an increase in the osteoporosis/osteopenia rate in the severe coronary lesions group. Multivariable logistic regression analysis showed that osteopenia/osteoporosis at the femoral neck was associated with an increased risk of developing severe coronary lesions. The multiple regression model showed that T-scores were the independent predictors of Gensini score. Most previous research, if not all, including our results indicate that low BMD not only were associated with increased risk of CAD, but also were an independent predictor of severity of coronary lesions in postmenopausal women.

Although many hypotheses have been proposed to explain the correlation between osteoporosis and CAD, it has not been thoroughly understood.^16,21,22^ In spite of common risk factors of bone metabolism and cardiovascular risk (inflammation, dyslipidemia, menopause, hypertension, smoking, and diabetes mellitus), the possible influence of genetics and vascular calcification also exists.^23,24^ Hydroxyapatite, an important part of the mineral phase of bone, is also found in the artery calcified plaque. Moreover, it has been reported that bone matrix proteins such as gla protein, bone morphogenetic protein-2, osteocalcin, and collagen were found in calcified plaques. Studies have suggested that some important gene mutations can lead to the early development of AS and osteoporosis, which indicate the evidence of common genetic basis.^25,26^ It’s worth noting that current evidence linking osteoporosis and CAD is far from conclusive. So, further study is needed to explore the relationship between the two common diseases.

### Limitations

The main limitation of our study is that the sample size is relatively small. Further studies involving a larger number of menopausal patients are needed to establish and confirm the relationship between the severity of coronary lesions and osteopenia/osteoporosis. In addition, information on impaired vitamin K status, inflammatory cytokines, gla protein, and osteocalcin, which might be associated with both coronary lesions and osteoporosis, was not available for this study. Also, the fact that the BMD tests were not performed in the same service was another limitation of our study.

## Conclusion

In this study, we investigated the association between BMD and severity of coronary lesions in postmenopausal women. Our results suggested postmenopausal women with low BMD are at high risk for severe coronary lesions. Future research should investigate common pathophysiological pathways between osteoporosis and severity of coronary lesions.
